# Thermoresponsive Gels

**DOI:** 10.3390/gels3010004

**Published:** 2017-01-10

**Authors:** M. Joan Taylor, Paul Tomlins, Tarsem S. Sahota

**Affiliations:** INsmart group, School of Pharmacy Faculty of Health & Life Sciences, De Montfort University, Leicester, LE1 9BH, UK; tomlinspaul@gmail.com (P.T.); ssahota@dmu.ac.uk (T.S.S.)

**Keywords:** thermoresponsive, micelle, hydrogel, organogel, UCST, LCST, multi-stimulus, drug delivery

## Abstract

Thermoresponsive gelling materials constructed from natural and synthetic polymers can be used to provide triggered action and therefore customised products such as drug delivery and regenerative medicine types as well as for other industries. Some materials give Arrhenius-type viscosity changes based on coil to globule transitions. Others produce more counterintuitive responses to temperature change because of agglomeration induced by enthalpic or entropic drivers. Extensive covalent crosslinking superimposes complexity of response and the upper and lower critical solution temperatures can translate to critical volume temperatures for these swellable but insoluble gels. Their structure and volume response confer advantages for actuation though they lack robustness. Dynamic covalent bonding has created an intermediate category where shape moulding and self-healing variants are useful for several platforms. Developing synthesis methodology—for example, Reversible Addition Fragmentation chain Transfer (RAFT) and Atomic Transfer Radical Polymerisation (ATRP)—provides an almost infinite range of materials that can be used for many of these gelling systems. For those that self-assemble into micelle systems that can gel, the upper and lower critical solution temperatures (UCST and LCST) are analogous to those for simpler dispersible polymers. However, the tuned hydrophobic-hydrophilic balance plus the introduction of additional pH-sensitivity and, for instance, thermochromic response, open the potential for coupled mechanisms to create complex drug targeting effects at the cellular level.

## 1. Introduction

During the past decade or so there has been interest in gelatinous materials (and their precursors such as xerogels) that respond to a change in their local environment e.g., specific ligands, pH and temperature among others, some of which are being considered as exploitable in, for example, advanced drug delivery systems. This represents a step change from inert polymer systems where the mechanism for delivery was passive diffusion through or dissolution from a matrix or reservoir. Of these materials, the focus here is on gels with thermoresponsive properties [1,2,3].

Gels are soft semi-solid materials that consist of components including those that act as the liquid dispersion medium (hereafter the “solvent”) and the gelling agent (gelator) of which the former is generally numerically the greater [4]. The distinction between thickening and gelling is often taken to be rheologically defined as the case when the ratio of loss to elastic modulus values (tan delta) is less than unity for a gel [5] but colloquially, soft masses formed by aggregates, as discussed below, are often referred to as gelling phenomena. The solvent molecules penetrate a hydrocolloidal network formed by the gelator, which itself can be of several types, some imparting elasticity while others, including inorganic particulate gels, easily permit disruption. Some gels, as in hydrogels, can crosslink physically or be covalently cross-linked [5,6,7,8] in which case, as in this review, the crosslinking agent can be seen as a gelling agent. Other systems can have a mixture of transient and permanent bonding e.g., lightly covalently stabilised with receptor-mediated glucose responsive gels [9]. Bridging molecules and particles create two- and three-dimensional aggregates of various structured types such as micelles (as discussed below) that are very viscous so-called gel phases (irrespective of tan delta value). Consequently, they depend on characteristics like molecular size, shape, relative hydrophilicity and ionic nature that maintain solvation. The latter is critical and opposes processes of crystallisation, precipitation and collapse, some of which can be reversible and form the basis of stimulus response. The viscoelastic characteristics of such materials depend on influences on both the solvent and gelator, but the permeability is strongly influenced by the usual factors i.e., partition (if the solvent is not water, for example) and the diffusion coefficient [10]. The latter depends on the microviscosity (i.e., the solvent), the interactive binding forces from the gelator and the porous nature of its resulting network. For the latter, both the extent (porosity) and the tortuosity are important especially for diffusing molecules approaching the size of the pores. The latter can change if the factors controlling the inter- and intra-molecular gelator junctions are affected by ambient conditions and this can also be exploited for responsiveness.

Not surprisingly in terms of biocompatibility, many thermoresponsive pharmaceutical gels consist of hydrophilic polymers in aqueous systems, although there are examples of apolar systems [11,12,13]. In either case, self-assembly structures including clays and polymers, combine physical weakness with a dynamic equilibrium that allows change and repair and like the solvation status, this has relevance to the responsiveness of the material to stimuli. By comparison, hydrogels and thermosets have a reinforced inter-chain structure that is usefully stronger but will not reform in many cases, if breached. Some non-rigid covalently bonded structures, however, are well known to be capable of stimulus responses because of elasticity, the ability to exchange solvent with the external environment or because responsive components have been designed in. In addition, there is increasing ability to fine tune the responsiveness to specific temperature tolerances [14]. New materials include dynamic covalent bonding and vitrimers of aqueous and non-aqueous type, some of which are within the scope of this review being composites with interesting intermediate characteristics conferring an ability to respond to temperature change [15]. The physical form of gels encompasses bulk and particulate materials including organogels, fast-responding nanogels, aerogels, xerogels and cryogels [16], which are peripherally included in this review, but may not always be biocompatible at an in vitro stage of development.

Sensor and actuation properties occur in gelatinous materials as uniquely simultaneous events. Physical and chemical influences provoke interactions involving solvent and gelator molecules. This can achieve a variety of results including viscosity change, current generated by redox, colour conversion and allosteric transformation in receptors. Utility can be found in controlled delivery of effector molecules [17] but also, for example, in healing [18], regenerative medicine [19,20], tissue engineering [21,22] and smart encapsulation of cells [23]. Chemo-electrical and gravi/volumetric differences can be coupled to accomplish work [24] and there are examples in, among others, responsive muscle-like contracting materials [25,26].

It has already become possible to achieve spatial targeting to tailor treatment by highly specified drug and gene delivery [26,27,28,29] via active docking by molecular partners at cellular and subcellular levels. The epitome would be the continuous and detailed response of materials to body, tissue or cellular changes in an imposed homeostasis that revolutionises treatment for diseases that continually metamorphose. One could imagine not only the accurate closed loop delivery of medication in diabetes and other conditions by using ligand-specific materials, but also the meticulously apposite auto-adaptation to clonal changes in metastatic disease.

There are recent reviews that cover aspects of the biomedical use of variously smart materials [30,31,32,33]. The way forward, as captured in the examples given in this article, is to use new synthetic, analytical and biological techniques to increase the robustness, cyclic reliability, precision, appropriateness and speed of response. Sometimes responsiveness can be achieved by maximising the surface area using nanostructures and so these aspects will also be included here, with the focus being on thermosensitivity singly or in combination with other traits.

## 2. Thermosensitivity, Thermoresponsiveness and Phase Transition Rheology

Many polymers, including natural ones like xanthan gum, starch, gellan, konjac, carrageenans, collagen, fibrin, silk fibroin, hyaluronic acid and gelatin [34,35,36,37,38,39], form gels by several main mechanisms as discussed by Gasperini [40]. These are often usefully temperature sensitive in terms of viscosity change, but thermoresponsive design will increasingly be combined with synthetic features that relate to specific monomeric inclusion that may transduce to a thermal signal or act independently. However, innovative types are also often thermally responsive in terms of outputs other than viscosity or volume, examples being opacity, colour (thermochromic), hydrophobicity and electroconductivity [41,42,43,44]. While viscosity changes underpin this review, these other features will be mentioned where relevant. The speed of response often depends on the physical form, principally the surface area and thus much emphasis can be found on nanoforms and this will also be explored. Thermoresponsiveness as a term is used to imply a critical change over a small temperature range, as opposed to a progressive thermosensitivity [45]. Honed individuality is widely represented in the literature and, in general, the variety of polymers with thermoresponsiveness is now huge [1,31,46]. In common with smaller molecules, polymers can exhibit not only glass transitions, but phase separation behaviours, at low and/or high temperature, somewhat confusingly known as Upper and Lower Critical Solution Temperatures, respectively. These transition lines distinguish solution the phase from undissolved two-phase systems that can cause a useful subsequent action. Values for these parameters are variously tabulated for a range of substances including polymers [47,48], with Aseyev focusing very comprehensively on non-ionic structures [49].

### 2.1. The Upper Critical Solution Temperature (UCST)

**Figure 1:**
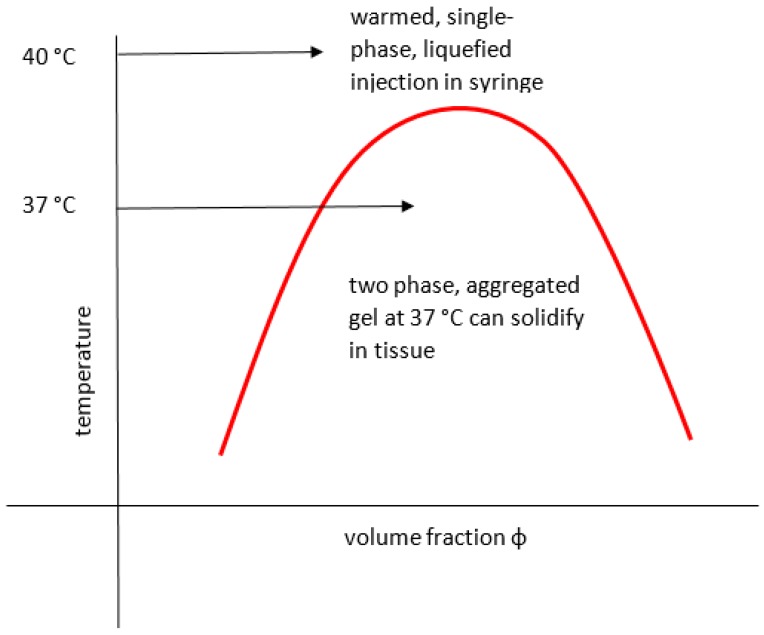
Gelling of injection below its upper critical solution temperature (UCST).

One such transition is the UCST which can be designed in to occur within a zone around body temperature and the effect on, for example, viscosity can therefore be exploited for medicine, including personalised drug delivery [50] sensing and feedback systems [51] as in Figure 1 that depicts an injection solution that gels on cooling to below a UCST designed for close to body temperature.

**Figure 2:**
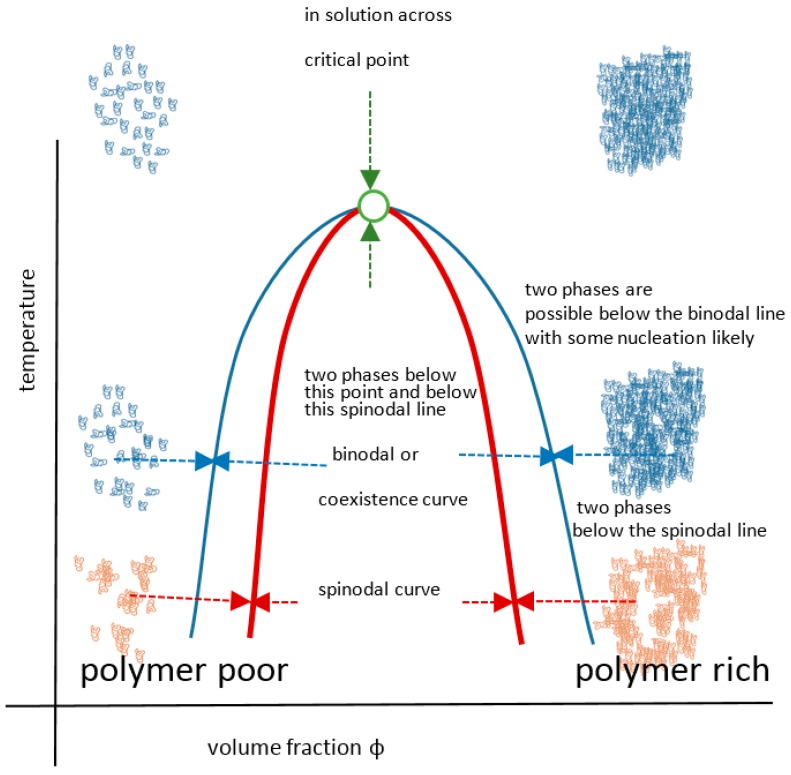
The UCST detail showing spinodal and binodal curves.

The UCST profile on a temperature-composition (volume fraction) plot for a polymer or blend is the less commonly documented transition type and is defined by the gelator coming out of solution *below* values on a critical separation line known as the spinodal curve, where in addition, a sensitive corridor exists between that and the so-called co-existence (or binodal) curve (Figure 2). These two curves are coincident at their apex, the critical point that represents the infinitely large molecular weight. This is often erroneously termed the cloud point, which is the temperature at which a sample becomes opaque at *any* concentration point on the critical curve. There are two mechanisms for phase separation, namely spinodal decomposition and nucleation plus growth. In practice, phase separation is measured by cloud point, but using turbidity as the criterion depends on the temperature change kinetics, sample geometry, analytical technique (e.g., light, neutron or x-ray scattering), the contrast between the two components, as well as viscosity and flocculation physics. Haas published his 1970 work on both poly(*N*-acryloyl glycinamide) and gelatin, where the Flory–Huggins and Flory–Rehner theories were applied to the study of the rubbery state of these UCST-type materials to gain insight into their crosslinking mechanisms. Poly(*N*-acryloyl glycinamide) was also used by Boustta for murine in vivo systems with controlled release over two to three weeks, of neutral and ionic drugs exemplified by model materials of a variety of molecular weights [52,53]. It should be clarified from the outset that there is a difference between in vitro demonstration of pharmaceutical potential in terms of function or biological engineering and the translation to a working medical product for which the safety, efficacy and advantage must be paramount, as discussed by Vert [54]. Safety and medical relevance have several facets including being compatible in pharmacological and inflammatory terms, the latter itself being divisible into molecular, morphological and topological aspects. For example, aggregated and fibrous polymeric forms such as carbon nanotube gels, popular for in vitro study [55], could be toxic in vivo because of biological processes [56,57]. Likewise, implanted materials will normally induce some degree of inflammatory response [58] related to a complex set of responses involving protein deposition, myeloid cell recruitment and fibroblastic walling off. The efficacy of drug delivery is related to the whole body pharmacokinetic constraints and also to any changes imposed on the pharmacodynamic properties of the drug carried, an example being the release of drug from covalent bonding to a carrier. These complexities are not always well mimicked by non-biological systems; however, the direct relevance difficulties they pose do not negate the value of simple systems to study mechanisms in vitro, on the basis that complexity may need to be built step by step for understanding. Consequently, the investigation of a wide range of polymer systems even outside biological tolerances is needed for progress.

**Figure 3:**
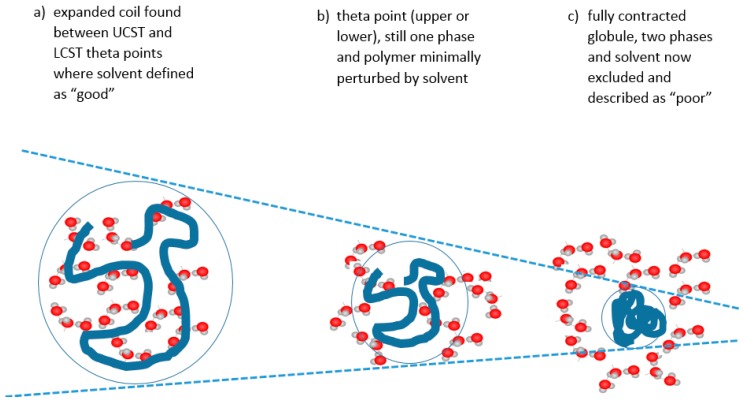
Relationship of the polymer form with temperature for a polymer showing UCST behaviour.

In colloidal solution, in a so-called “good” solvent, the polymer molecule is in a loosely coiled state (a in Figure 3). This state exists between the LCST and UCST theta points, where this range can be demonstrated, as explained later in this article. For the UCST case, when cooled to the theta point (b in Figure 3), the solvent passes through the point of minimal polymer solvation as it tends towards “poor” (c in Figure 3). At this latter stage, the polymer molecules and the solvent are enthalpically driven to associate preferentially with themselves, as quantified by the Flory χ parameter. Solvation then fails, taking the system below the UCST curve.

The assumption is that the UCST is above the freezing point of the solvent, and aqueous systems displaying UCST have therefore not often been found, though this may change with advances in polymer design. Where this two-phase state can be demonstrated, i.e., a compacted polymer sphere that is by definition not interacting with solvent, the viscosity might be expected to reduce. However, hydrophobic aggregation may instead produce interesting gelling systems (see below).

The solvated polymer configuration is assumed to move randomly within the confines of a spherical locus but the model is unlike other random walk simulations because it cannot involve time-space superimposition. Therefore, in predictive models, the theoretical volume of the contracted but soluble “globule” model has had to be increased to allow for this.

**Figure 4:**
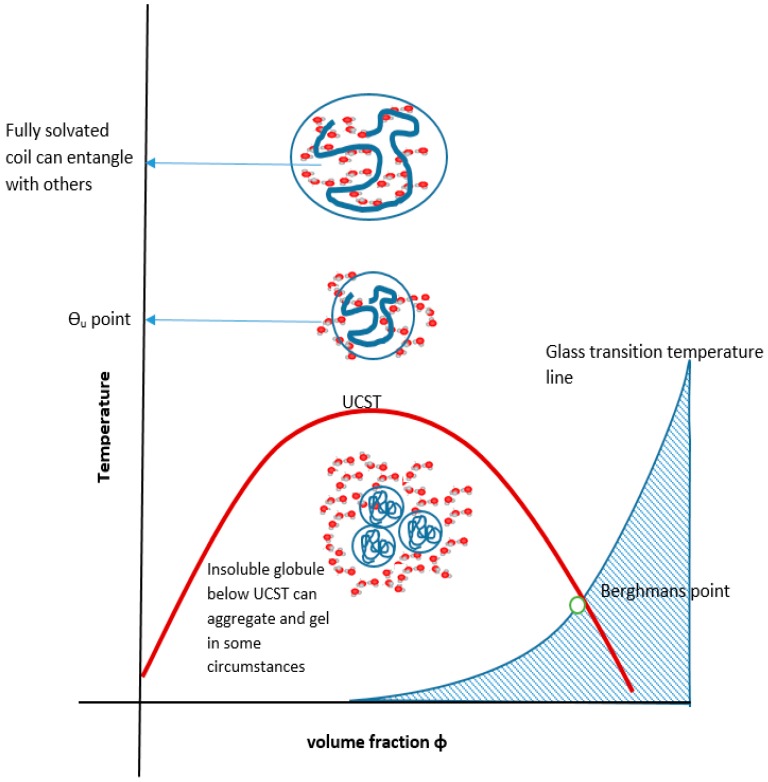
UCST, glass transition (Tg) and Berghmans point.

Cooling the coiled molecule towards the critical curve (but above Berghmans point (BP) on the glass transition (Tg) curve (see Figure 4)) first reaches the so-called “upper” theta point (Ɵ_u_), as defined by Flory for polymers, which lies above the spinodal curve.

Using the Mark Houwink plot of log molecular mass vs. log intrinsic viscosity, η allows the determination of the exponent α which takes a value of 0.5 at the theta point Ɵ_u_ [59]. At ϴ_u_, the entropic solvent swelling effect on the polymer is equal and opposite to the enthalpic energy of polymer-polymer interaction which is a contracting influence. The detail of the thermodynamics have been reviewed elsewhere [60,61].

Above the ϴ_u_ temperature value, interactions between extended, solvated coils usually form larger structures especially if branched, relying on van der Waals, electrostatic, hydrogen and occasionally metal coordination bonding [62]. Inter-chain junctions comprise entanglements and knots [60]. At rest, entanglements are in dynamic equilibrium but maintain a three-dimensional assembly imposing a viscoelastic status detected by oscillatory rheometric techniques. They are easily disrupted by rotational stress, often showing a pseudoplastic drop in viscosity with increasing rotational frequency at a given temperature. Further heating of the extended coil pushes the system towards LCST behaviour (see below). Materials may exhibit a true UCST, above which the solvent and solute phases are miscible but, since other phase changes are recognised, the temperature (T) polymer volume fraction (φ) profile should be numerically described before applying the UCST and LCST terms [31].

**Figure 5:**
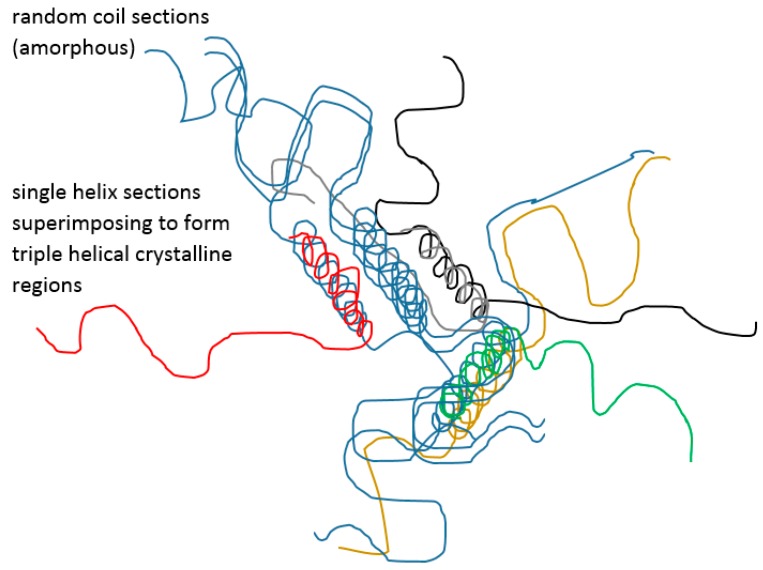
Gelatin coil and helix ‘crystalline’ regions.

Particular materials, such as gelatin and agarose, deserve attention in the discussion of UCST. Gelatin has long been used in a variety of pharmaceutical products, ranging from the simple suppository which is required to liquefy at body temperature to skin preparations which may be required to solidify after applying the melt. Regardless of how familiar these are, with gelatin featuring strongly in recent biomedical research, such as nanofibres and copolymers, and despite well-established observations and facts, including those of Haas [52,63,64], there remains confusion about the thermal behaviour mechanism. It is well known that cooling a hot concentrated gelatin melt allows single and then triple helices to be formed between coils (Figure 5), in the gradual desolvation process [65,66,67] as the solvent becomes effectively “poorer.” This means that, as in the case of simpler coil to globule-forming polymers, the polymer-polymer chain interactions gradually outweigh those of the solvent-polymer, whereby solvent is progressively excluded and a helix results for gelatin. The helices become further intertwined to resemble the parent collagen and the resulting junctions predispose to some crystallinity within the larger network that may retain considerable amorphous regions [68]. For gelatin, at high concentrations (>15% *w*/*w*), an elastic gel is therefore formed around 35 °C, depending on several factors including concentration and thermal history. Viscosity hysteresis in a heating-cooling cycle indicates the time dependence of the solid structural arrangements so that fast-cooled gels will contain mainly amorphous material, whereas a slowly cooled system allows the helical rods to align to form distributed crystalline regions. Badii established some years ago that for slowly formed solid gelatin thermograms recorded through reheating indicate a mobilisation of amorphous material in addition to a melting endotherm for the present crystalline regions. Conversely, the latter are missing from a fast-cooled sample [68,69]. Haas reported that gelatin and poly(*N*-acryloyl glycinamide) (PNAGA) crosslinking and solidification differed in that crystallinity was a major feature only in gelatin [52], thereby adding weight to the idea that gelatin does not display a real UCST. However, gelatin cooling through the solidification point is often termed a UCST phenomenon perhaps analogous to heating poly(*N*-isopropylacrylamide) (PNIPAm) above the LCST [70,71] as is discussed later. Interestingly, although the helix formation is part of the progressive desolvation of the gelatin coil, in general the temperature journey to the ϴ value dense sphere, towards either LCST (temperature rise) or UCST (temperature fall), means that the coil form and attendant solvent interactions are minimised. This may not be the confirmed case for the helices at the gelatin solidification temperature across the gelling concentration range. In gelatin solutions that were chosen to be diluted to gel, i.e., 0.25 to 1% *w*/*w*, the cloud points (referred to as both Flory or ϴ_u_ temperatures, despite clearly not being at an apex concentration) were reported by Gupta to be at about 15–17 °C, i.e., much lower than gelling temperature. Gupta was unable to demonstrate an explicit UCST where insolubility occurred [72] and it therefore seems yet to be clarified if the gelation at higher concentrations is a glass transition or a UCST.

Seuring points out that although in general UCST materials have been identified less often for medical use than LCST (see below), there is room for design and trial of promising new polymeric candidates [73]. The UCST condition is enthalpically driven, as explained above, deriving from strongly exclusive bonding within the solvent and within the polymer. In general, polar materials displaying explicit UCST-related cloud points can be divided into several types, including HB-UCST that rely on H-bonding, those with supramolecular crosslinking additives and C-UCST that involve Coulombic forces. The latter include zwitterionic interactions and relate to ionic materials and milieu [73]. To be widely useful, the working range for a UCST should arguably be in the 0–60 °C range (narrower if for tissue use), operate at low ionic strength, be predictable, reversible, repeatable and sharp. It should be independent of concentration, counterion or pH, suggesting that HB-UCST materials may be more relevant. However, development has been slow and Seuring cites cases such as the previously mentioned H-bonding PNAGA, for which ionic sensitivity is so great that its UCST is often suppressed (and was long unrecognised) due to acidic impurities active at even low concentration [73]. Approaches to producing polymers useful for their USCT were to minimise or neutralise ionic groups, maximise the H-bonding capacity, design against hydrolysis and use copolymers with regular repetition. Thus, for example, a tri-stimuli responsive injectable algin-aminocaproic acid thixogel that formed a sol on heating was recently reported by Chejara [74]. Maji presents a study of l-serine-based zwitterionic polymers with a UCST that is relevant for biomedical use, although the low pH would appear to be a potential problem in tissues for a depot dose [75]. Ji has looked at bespoke polypeptide UCST (and LCST) polymers for cell toxicity in the targeted delivery of cytokines [76]. Some examples that are not explicitly UCST but appear to be broadly similar in that heating produces a gel to sol transition, include work by Gao [77] who describes a low molecular weight phenylboronic acid material that in aqueous polyethylene glycol solution forms a fibrous gel when cooled to 37 °C. It was used with doxorubicin such that after intratumoural injection of the warm, liquefied preparation, this drug became encapsulated in the gel structure that formed in the tissues and could sustain delivery, maintaining effectiveness but reducing general toxicity compared to plain carrier.

Yao describes a fibrous supramolecular, multi-stimuli metal-organic gel system [78]. This is stabilised by hydrophobic and π–π interactions in addition to a coordination component with lead nitrate in low molecular weight benzimidazol ligands. The aqueous, thermoresponsive metal-organic gel formed a sol on heating, as well as being pH and chemically sensitive, and has been used to scavenge dyes from waste water. Conceivably, the same technology could be used in systems for removing poisons from plasma.

Examples of UCST behaviours can be found in polar and apolar solvent systems in a range of disciplines. The classical case of polystyrene in cyclohexane is discussed later in this review in a wider context, but some viscosity adjusters in automotive mineral oils displaying UCST-related phenomena may be transferable to wide temperature range equipment such as pumps, or to design of heat-sensitive organogels that might find application for drug delivery. Thus, engine oil formulation choices must be temperature tuned to prevent the polymer additive to fall below the UCST line where it would become insoluble. Around the theta point (ϴ_u_), one type of additive, poly(alkylmethacrylate), is a minimally oil-solvated globule that therefore does not significantly increase the viscosity of the oil. This is an advantage allowing the oil to assist in the cold starting of engines. At higher temperatures, the additive becomes better solvated and assumes a coil form. Each additive molecule is able to mesh with others and increase the viscosity of the oil so that it can lubricate and load-bear well at high temperatures. The oil itself will have a falling viscosity with rising temperature (normal Arrhenius relationship) but the fall will be reversibly mitigated by the expanding additive because of its globule to coil transition [79], thereby forming an impressive self-adjusting or closed loop system.

Organogels are gelling systems that relate to the above oil additive example, because they are often responsive with a UCST and thus fit within the current remit, having the possibility of biomedical use [80,81,82]. Organogels can be covalently crosslinked (see below with hydrogels), but again, as for the polar equivalent, they can be weaker, more transiently stabilised structures formed in dispersion sometimes from low molecular weight gelators [83,84] in apolar solvents that include hydrocarbons such as hexane, esters such as isopropyl myristate and vegetable oils, the latter being suitable for dosage forms. Low molecular weight may include the optically responsive (e.g., anthracenes) and respond to a heat source. These could be H-bond or comprise π-π stacking systems and can self-assemble in this way in vivo [85,86] and thus have imaging potential.

Some organogel formulations form a category that is intermediate with hydrogels, where there is a small proportion of water in the solvent mix. In these, nanotubular structures arise from reverse micellar networks filled with water to form polar channels [87]. Some organogels are not explicitly described as having UCST values but may reasonably be assumed to have similar phase transitions, such as in the following dually responsive cases. Zang describes [88] a novel multi-stimuli responsive organogel containing salicylidene Schiff base derivative (cholesterol 2-(3,5 di-tert-butyl-2-hydroxybenzylideneamino)acetate) that self-assembles into nanofibres that undergo a reversible gel to sol transition on heating, where additionally the gel but not the sol has strong fluorescence, associated with the fibre formation. The material is also zinc and possibly fluoride ion responsive [41]. Yang describes an azobenzene gelator in an apolar solvent that can be reversibly triggered by heating, UV excitation or by shear [88]. A dually active organogel system for injected thermoresponsive chemotherapy has also been proposed [89]. In this study, bridged pillarene dimers with a guest linker in the gel state can also emit fluorescence and thus act as a tissue-imaging agent when delivering temperature-targeted drugs as the triggered sol. These simple UCST-type organogels should be distinguished from LCST organogels, bigels and crosslinked organogels which are discussed later.

### 2.2. The Lower Critical Solution Temperature (LCST)

This is the more frequently encountered circumstance than UCST for polymers and their blends. When heating above the LCST, dissolved polymers like PNIPAm at their critical gel concentration (CGC) will exhibit aqueous insolubility, being above cloud points on the LCST curve where aggregation of desolvated polymers can occur [90]. There is an analogous theta point, the ϴ_l_ just below the LCST curve [91] as is reviewed below. The underlying process of contraction of the extended coil to a globule as the solvation decreases [92] on heating is documented for many polymers including, for example, dextran [59,93], and bears discussion in this context. The globular form depends to some extent on molecular weight, such that the typical hydrophilic polysaccharide dextran of molecular weight 200 kDa, is described as assuming a coil of classic Flory spherical shape in water. Above this molecular size for dextran, Masuelli notes that the extensive branching causes a conformational change to an ellipsoid with a detectably increased value of chain stiffness parameter (defined as log viscosity to temperature ratio, d(ln [η])/dT). The normal Arrhenius relationship whereby the dextran gel viscosity falls on heating towards ϴ_l_ is explained by the size and shape contraction as the solvent becomes again poorer and polymer-polymer interactions predominate (as was the case for the upper theta point ϴ_u_). Antoniou describes the contraction as entropic and dependent on the loss of solvent-related interaction with dextran OH and ether –O– hydrogen bond acceptor groups [59]. The Mark-Houwink α value tended towards 0.5 as the temperature was raised from 20 to 40 °C, again indicating the globule state. Güner had previously reported the ϴ_l_ for dextran to be 43 °C and stated that this was related to LCST behaviour [94], but it is also well known that polymers with an overwhelmingly hydrophilic character will not demonstrate an actual LCST. Other materials will not show water insolubility under normal pressure conditions e.g., if it is above boiling point [95]. Masuelli reported Mark-Houwink α values of *below* 0.5 for dextran heating which appear to suggest conditions between the ϴ_l_ and an LCST, but this anomalous value was evidently due to applying linear polymer modelling to a hyperbranching system as referred to above.

Aqueous polymers for which the LCST is below the boiling point of water can progress to insolubility on heating beyond their ϴ_l_ (analogously to the UCST case). This large group includes many well-known examples of pharmaceutically useful polymers such as a variety of substituted *N*-acrylamides typified by PNIPAm and some substituted celluloses and chitosans and the wider poloxamer family to name but some [96]. The most often reported of these, PNIPAm, is widely reviewed elsewhere and debated as to its biocompatibility. Two recent papers have reviewed and measured this in biological and ophthalmic systems and reported that toxicity, while measurable, may be cell-type and time dependent [31,97].

Li reports a cyclodextrin-poloxamer system with LCST suitable for medical application such as drug and gene delivery [98]. For non-crosslinked polymers with relatively hydrophobic groups, progressive exclusion of the water solvent on heating beyond the LCST creates an entropically driven hydrophobic self-association. In the case of PNIPAm this implies the isopropyl groups where some of the hydrophilic pendant amides project to interact with bulk water through the hydrophobic collapsed backbone and isopropyl core [99]. Thus the LCST, like the UCST depends, for linear and branched chains, on the dominance of hydrophobic groups [93]. The coil to globule transition including the dehydration of the coil and additional aggregation such as for PNIPAm has been variously proposed as both single and multistage models [30,100,101,102]. It should be noted that between the temperatures of 20 and 32 °C, PNIPAm shows the more typical Arrhenius viscosity reduction with transition from coil to globule before the sharp rise at the spinodal curve [103] where aggregation and, at high concentrations, gelling occurs as described. The literature does contain examples of LCST insolubility that occurs without gelling such as the case of threonine-based chiral homopolymers that precipitate reversibly but with profiles that are very pH dependent [104]. Normally, however, the process of the phase change is dependent on the conditions and the structural detail so that, for example, Costa looks at adjusting the LCST value of PNIPAm with salts and pH while Silva describes the similar behaviour of hydroxypropylmethylcellulose (HPMC) [101,105]. Shi discusses a PNIPAm biconjugate with azobenzene and rhodamine that upon irradiation changes conformation reversibly, thus modulating the coil-globule transition temperature as a result [106]. That all polymers with LCST insolubility and gelling behaviour undergo a slower rehydration when temperatures fall although the hysteresis is the subject of debate. The aggregation step as in PNIPAm was reported early as fully reversible, though reports to the contrary continue to emerge [107,108,109]. Jeong [110] long ago described the factors important for the kinetics of reversible systems of this sort. However, they are additionally dependent on the total surface area, so that subdivided systems such as micelles and nanogels re-equilibrate faster (see below for these). In one study with gold nanoparticles coated with PNIPAm, the rationale is explored for the conditions needed for the polymer to influence aggregation and the importance of excess PNIPAm [111]. Polymers that respond with heat-induced agglomeration-related viscosity rise are dubbed “negatively thermoresponsive.” The most useful candidates in the flurry of design possibilities for drug delivery are those that gel predictably near body temperature, so that materials can enter the body cold (rather than hot as is possible in UCST systems) and will gel at or before 37 °C. Therefore, when Yang discusses alginate beads with an LCST of 55 °C, these show potential but need modification to make the upper temperature achievable safely for the drug delivery proposed [112]. In a study that indicates how unpredictable design can be, Yu constructed core shell nanogels with opposite charges, using modified but thermosensitive PNIPAm. The electrical neutrality was independent of temperature and this system was proposed as a novel in situ gelling system [113].

Lastly in this section and in analogy to the UCST cases, there are simple (i.e., non-crosslinked) organogels with LCST-type behaviour, such as the dual-stimuli homopolymers of poly(7-methacroyloxycoumarin) that exhibit a tunable LCST separation in chloroform among other solvents [114]. Some organogels are structured particulates, such as the ciprofloxacin-carrying shell core stearic acid-alginate type described by Sagiri [115], are at least temperature sensitive if not defined explicitly as LCST.

### 2.3. Micellar Systems with UCST and/or LCST

Developments involve the self-assembly of multiblock copolymers with amphiphilic properties to form micelles with UCST properties as well as those with the more common LCST profile. Many of these produce thermoresponsive gelling systems that have biomedical prospect.

#### 2.3.1. Micellar UCST Systems

The synthesis techniques are now refined to an extent that numerically defined structures with thermoresponsive blocks and grafts can be produced to tune UCST polymers to required ranges (depending on proposed utility). These are extensively reviewed by others [49,116,117,118,119,120]. Fuijihara [121] describes micellar diblock copolymers with pendant ureido groups. These are in aqueous solution of the monomeric form above the UCST curve but assemble below it at 32 °C to form micelles with a core of poly(2-ureidoethyl methacrylate) or PUEM and shell of poly(2-methacroyloxyethylphosphorylcholine) or PMPC. In the general case, desolvated micelles may then further aggregate to form a non-covalent gelatinous network below the UCST transition, the gelling temperature depending on the hydrophilic-hydrophobic sections that form shell and core respectively. Ranjan [122] has returned to a well-studied surfactant Triton-X that consists of polydisperse preparation of isooctylphenoxy-polyethoxyethanols. Solutions of below 35% *v*/*v* are viscous solutions but at higher concentrations are homogeneous gels (35%–60% *v*/*v*) or heterogeneous melts (>60% *v*/*v*) with a previously unrecognised UCST separating the 35% *v*/*v* sol and gel domains at about 25 °C. The model here was a nucleation such that the nucleus radius was 5 nm in the gel phase below the UCST, but 4 nm in the solution and the supra-UCST sol region. Many other such micelle examples can be found, including the synthesis of a methoxy-poly(ethylene glycol)-*block*-poly(acrylamide-*co*-acrylonitrile) (mPEG-*b*-poly(AAm-*co*-AN)) amphiphilic copolymer tunable series with UCST, and tested in vitro for responsive delivery of doxorubicin on cultured tumour cells [123].

**Figure 6:**
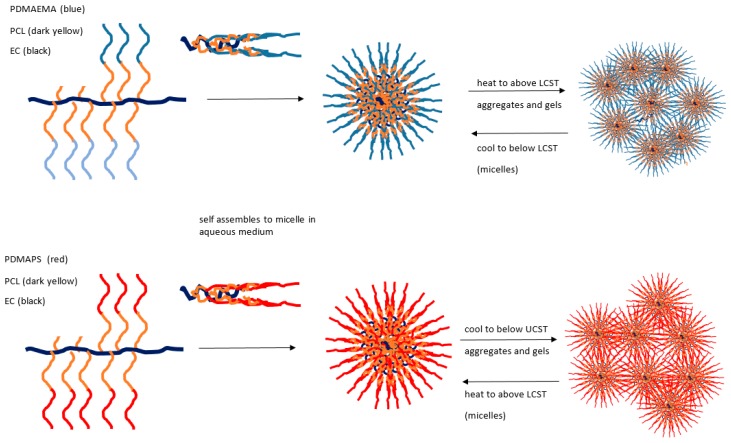
Related EC-g-copolymer aqueous micelle systems that gel when aggregated beyond LCST or UCST respectively. Adapted from [124].

Yuan [124] has reported the synthesis and characterisation detail of two series of ethylcellulose graft (EC-g-) copolymers showing opposing thermoresponsive behaviours. Structurally, each ethylcellulose backbone has a graft comprising a poly(ε-caprolactone) (PCL) component in a block structure, the first with poly(2-dimethylaminoethyl methacrylate) to form EC-g-(PCL-*b*-PDMAEMA) and the second, a quaternised version, with poly[3-dimethyl(methacryloyloxyethyl) ammonium propanesulfonate] to form EC-g-(PCL-*b*-PDMAPS). Of these two, the first demonstrates an LCST, while the quaternised structure gives a UCST response (see Figure 6).

For the EC-g-(PCL-*b*-PDMAPS) system, a sharp increase in transmittance and a sharp decrease in hydrodynamic radius was observed above 32 °C. Thus, unlike the conventional coil to globule shrinkage in some UCST systems, interactions of many micelle types are revealed by size expansion at temperatures below the UCST and gels may form. In the EC-g-(PCL-*b*-PDMAPS) case, quaternised groups collapse as expected but form aggregates of this kind, whereas above the transition temperature, extended micellar coils form analogously to the conventional model. An opposite case with LCST for the non-quaternised version is also described in this paper.

Because of the restrictions in aqueous design of UCST systems in general, as referred to above, binary solvent mixtures have been proposed such as the ethanol-aqueous systems discussed by Zhang [96]. These create possibilities in areas not possible in totally aqueous systems and any additional toxicity of ethanol is justified by Zhang as remaining suitable for medical and personal care products in view of the advantage of how finely adjustable such systems can be. He reviews the underlying theory of cosolvency and non-cosolvency as well as several examples of micellisation in the ethanol-water system such as versions of poly(methyl acrylic)-*b*-polystyrene (PMA-*b*-PS) block copolymers with a PS core and UCST values above that of PMA itself. The importance in the physical manifestation of thermal transitions is demonstrated by the double hydrophobic polystyrene_88_-*b*-poly(methyl methacrylate_80_) (PS_88_-*b*-PMMA_80_) polymer that has been developed. It also self-assembles at low concentration in 80% ethanol into micelles. However, at higher concentration (1%), it gels as a UCST response due to proximity of the large radius of gyration for PMMA structures [125].

#### 2.3.2. Micellar LCST Systems

At the critical micelle concentration (CMC), raising the temperature again dehydrates the structures by destabilising the equilibrium hydrogen bonding that favours the micelle. This fosters increased hydrophobically stabilised entanglement in the micelle core which contracts, typically leaving hydrophilic groups in the corona to interact with the aqueous solvent. The relative hydrophobicity of the structure determines the CMC and the LCST (often referred to as the Critical Micelle Temperature or CMT) [126,127]. Above the latter, the polymeric solute comes out of solution but may simply maintain a collapsed micellar state. The more dominant the hydrophilic component, the higher the LCST, although some less hydrophilic components may be subjugated to allow particular moieties such as PNIPAm to have a dominant effect on the LCST [27] and subsequent gelling of an aggregated micellar system. Not all insolubles may aggregate and gel [128] but in general this LCST behaviour has the appearance (but not the thermodynamic drive) of being a mirror image of the micelles discussed above with UCST properties and has the general advantages of LCST systems.

A classic example of micellar LCST behaviour is the poloxamer group, patented in the 1970s. These triblock amphiphiles are based on polypropylene oxide and polyethylene oxide (PEG_2-130_-POP_15-67_-PEG_2-130_). Poloxamers form micelles by internalising the relatively hydrophobic PPO segment and, though often described as spherical, may be cubic, cylindrical or elongated (worm-like) depending on concentration among other factors. Poloxamer F-127 has an LCST close to body temperature and has been applied to drug delivery and a variety of other medical uses such as nanoparticle coatings, cell targeting with cytotoxics and delivery of phototherapeutic agent like chlorin e6 [129]. Gelling makes them potentially useful for localised injection and for cavity instillation [127,130]; much as has been suggested for PNIPAm, so that solutions of around 20% *w*/*w* can be syringed when cold and can be used to fill wound cavities as conforming liquid bandages that solidify in situ. These have been proposed as delivery agents for local anaesthetics, cytotoxics, antibiotics and anti-inflammatories.

Despite the interesting properties of poloxamer, many alternative micellar systems have been proposed because, for example, one of the problems with poloxamer and similar structures such as tetronics is that the gel phase forms at relatively high concentration (20%–30%) [106]. Poloxamers are described as erodible but not biodegradable, and so concentration is a concern among other potential health issues that have been well recognised for many years [131]. Since that time, a variety of well-defined systems have arisen of which the following are examples. A polylactide-polyethylene glycol-polylactide (PL-PEG-PL) series was synthesised in a one-step procedure that avoided toxic coupling agents. It clearly has similarities to poloxamer [132] but unlike poloxamer, was biodegradable, though performed for several weeks before being cleared when trialled as an anti-adhesion cavity filling protective for bowel injury treatment. Micelles underwent phase transition to a gel within 2 min in vitro and in vivo and compared well with hyaluronic acid gels described as standard for the purpose. Hydrogel and organogel formulations with polylactide have been reviewed [133] and include the covalent and non-covalent use of d and l stereoisomers with glycolic acid in copolymers with PEG, polycaprolactone, polyurethane and others.

The commercial use of polylactide has been reviewed [120] and includes Oncogel^®^ which is a PL-PEG-PL formulation as that considered above with co-formulated paclitaxel for injection as a sol that undergoes conversion to a gel depot.

Chitosan is a low toxicity, natural water soluble copolymer of the saccharides β-(1-4)-linked d-glucosamine and *N*-acetyl-d-glucosamine and is the partially acetylated form of insoluble chitin of crustacean shell origin. It is protonated in acidic milieu and can be quaternised in the trimethylamine form. It is not naturally thermoresponsive but in combination with other materials can become so and thus chitosan-β-glycerophosphate systems are widely reported [134]. In the present context, several examples can be found in recent literature of chitosan copolymers used to form micelles with an LCST. Thus, for example, grafting chitosan to PNIPAm produces nanomicelles used with a payload of curcumin, the LCST being tunable between 38 and 44 °C [135]. A triblock graft polyacrylic acid (PAA) copolymer series of phthaloylchitosan-PNIPAm-PAA has been prepared by RAFT polymerisation [136]. These polymers form micelles that have LCST transition temperatures that are adjustable between the crucial temperatures of 34–40 °C by chain length, branching, pH and concentration, with the sharp, reversible transitions at pH 4. Other chitosan-containing micellar systems have sensitivities to other stimuli, such as redox [137], and are mainly outside the scope of this review.

Cellulose-based materials, e.g., hydrophobically modified hydroxyethylcellulose (HMHEC), can form nanomicelles with a hydrophobic core loaded with the light-emitting reporter poly(9,9-dioctylfluorene) [138]. As explained earlier, it cannot be assumed that materials that have a micellar LCST and thus lose solubility above it, will necessarily gel [139]. In the HPC-g-PDMAEMA case, above LCST and at high pH, the micelles seem to destabilise rather than further aggregate and gel. Chen also reports a triblock (polycaprolactone–PNIPAm-β-substituted alanine) non-aggregating micelle system which was used to carry doxorubicin and the light-activated material meso-tetraphenylchlorin (m-TPC). This is thermoresponsive with an LCST that was tunable with the introduction of the pH-sensitive moiety (alanine) because of the protonation of the carboxylate groups. The proposal was that at body temperature and neutral pH, the micelles would be stable, but in the mildly acidic tumour environment, the structures are merely destabilised and this allows drug release [140].

Micellar systems in general are widely featured in the recent literature partly because of the increasing ability to synthesise their component complex polymers. Particular micelle synthesis designs for LCST are attributable to He [141] who describes a facile, one-pot RAFT polymerisation method to create block copolymers of pentafluorophenyl acrylate (PFPA) and methyl salicylate acrylate (MSA) esters that form dually responsive micelles. He reports utilising the ester selectivity towards a series of aliphatic amines to modify the backbone with amino groups while maintaining the block shape. This elegant investigation produced micelles that were pH and temperature responsive, indicating the increasing ability to design smart structures. However, in this particular case, with LCST values around 65 °C, they were not directly suitable for in vivo use because the aim should be to inject coolly and for body temperature to gel the formulations. These investigative studies pave the way for more directly relevant materials with respect to tissue use. Similarly, other problems centre on solubility issues such that Kim has designed a dually responsive micelle that is composed of a PNIPAm chain with random single inclusions of malachite green that confer photoresponsive qualities, potentially suitable for imaging and possibly phototherapy, in addition to the LCST. The micelle can aggregate with rising temperature but, due to the low solubility of the complex below the LCST, the concentration restraint may have somewhat inhibited gelling in this particular design, inviting developmental modification [142].

**Figure 7:**
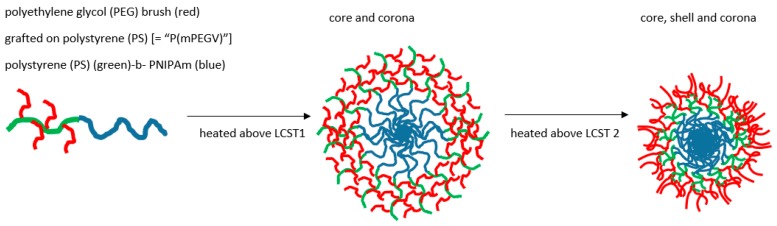
Dually responsive (i.e., two sequential stages LCST) micelle formation from poly(mPEGV_466_)_18_-*b*-PNIPAm_60_ in water. Adapted from [145].

Landmarks in thermogelling micellar design include the characterisation of systems where, for example, Weiss showed micelles progressing to further aggregation via two LCST-related cloud points where contraction of the core occurred at the lower temperature LCST, whereas multimicellar aggregation took place above the second [143]. A decade before, Xu had shown a RAFT polymerised NIPAm-PDMA micelle system had a similar accretion stage associated with a second LCST, that could be opposed by dense branching [144]. However, in another complex RAFT product, temperature-induced core-corona and core shell corona stages have been documented as associated with the multiple critical temperature behaviour. A copolymer was thus formed of PEG and a vinylphenyl (V) component to give rise to a diblock section (mPEGV) comprising short PEG brush on an essentially polystyrene (PS), i.e., the pendant phenyl on linear backbone (see Figure 7). This was then further bonded to a terminal linear PNIPAm to give poly(mPEGV_466_)_18_-*b*-PNIPAm_60_ [145].

On raising the temperature through a first and second LCST value above which gelling may occur, the preliminary collapse was of the PNIPAm core producing a corona of PS-PEG, followed by the second of the PS moieties leaving a corona of PEG surrounding a PS shell enclosing the original core of PNIPAm.

Liposomal particles are superficially similar to micelles and are briefly included here. They have a bilayer and more permanent structure than the dynamic nature of micelles. In the simple spherical form, at low concentration, they can be constructed as unilamellar or as a set of concentric lipid bilayer walls assembled typically from a tightly packed lecithin derivative. The lipids are capable of undergoing gel–sol transitions which can be tuned to just above body temperature. However, the term “gel” is being used here in a different sense than for polymers and micelles, in that the region inside each bilayer at temperatures below a transition becomes less mobile. Thus, gelling occurs within the nanodimensioned bilayer and is therefore separate from most of the examples tackled in this review. However, it is also well known that for phospholipids and the like, the relationship between micellar, lamellar and non-lamellar lipid phases involves agglomerates, such as the hexagonal and cubic liposomal forms that overlap with the gelled micellar amphipathic polymers discussed above [146].

### 2.4. Materials Displaying Both a UCST and LCST

**Figure 8:**
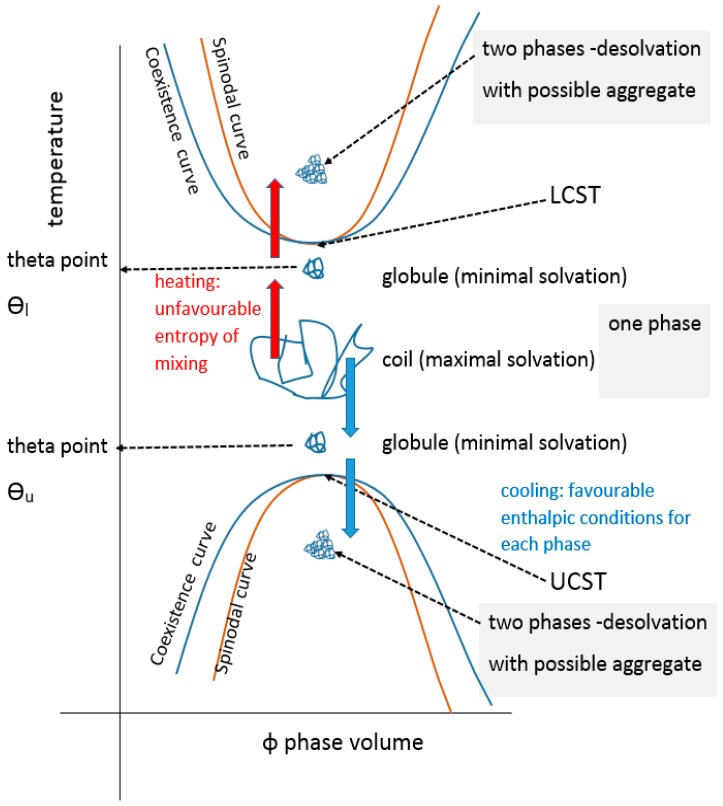
Unified LCST and USCT showing both theta points.

All partially miscible polymer liquid or polymer/polymer mixtures may have, at least in theory, both a UCST and an LCST, there being a theoretical continuum (Figure 8). In practice, they may not be identifiable experimentally.

**Figure 9:**
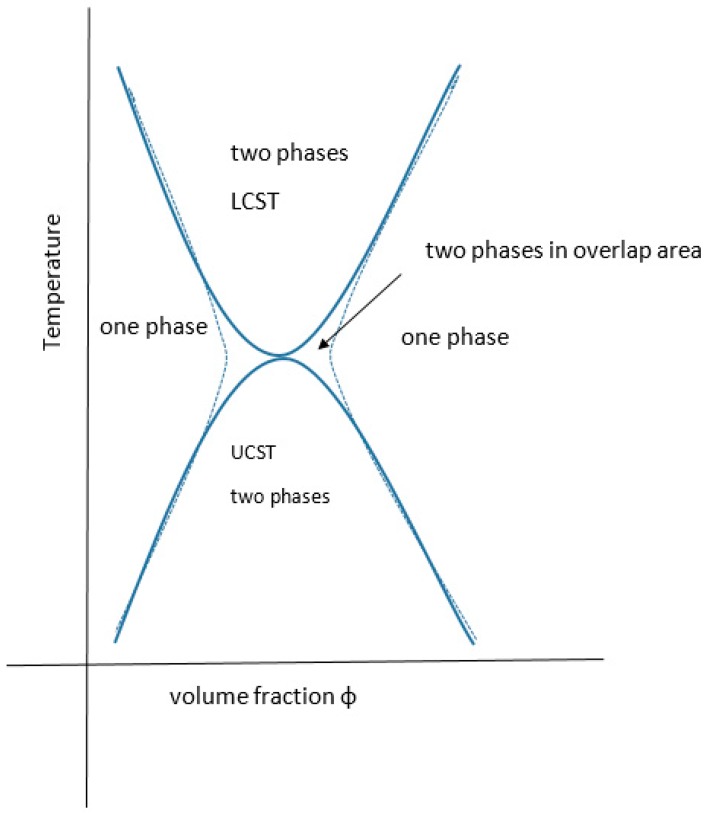
Hourglass pattern of some combined LCST and UCST behaviours.

There are many examples of mixtures where the LCST has been calculated to be above the degradation temperature of one or more of the components and for many other systems the theoretical UCST is below the glass transition temperature of one or more of the components. There are also many examples where the kinetics of phase separation are too slow to monitor experimentally due to the high viscosity of the system. This might be resolved by adjusting the conditions (e.g., molecular weight, temperature, pressure, ionic moieties or environment, pH or valency of coordination ions). The general case for materials displaying both critical regions has been fully reviewed recently by Clark and Lipson who present several theoretically related phase diagrams, including the extreme where the upper and lower critical solution temperatures are superimposed or where, with poorer solvents, the curves are joined in an hourglass pattern of immiscibility, for critical pressure and molecular weight values [92] (see Figure 9). The systems presented by them are organic solvent-based including a star-variant of PS in cyclohexane. Both theta values (ϴ_u_ and ϴ_l_) have been reported for a few materials [147]. As far back as 1971, the subject was addressed by Siow for polystyrene in acetone and polyisobutylene in benzene [148] and yet there is sometimes confusion between the Flory ϴ_u_ and the theta type equivalent ϴ_l_ for LCST. Aqueous systems that exhibit both are rarer than inorganic solvent-based ones, some like PEG actually display a closed solubility loop. The polystyrene in the cyclohexane system is the archetypal example with the theta points found at 33 and 210 °C [148], where the polymer has assumed the compressed sphere, there being an extended coil between these temperatures. More UCST cases are, however, emerging and Seuring points out that the aqueous solutions of poly(vinyl methyl ether) (PVME), poly(vinyl alcohol) (PVA) and poly(2-hydroxyethyl methacrylate) (PHEMA) display both UCST and LCST, although for these the UCST is below normal pressure water freezing point [73]. Zhu, however, reports the synthesis of a family of zwitterionic homo-, co- and terpolymers that have this property as the linear non-aggregate [149], although the systems may aggregate without truly gelling and are complex with some overlap as discussed elsewhere [92,150] (see also Figure 3). Balu reports a protein-polymer of the 16-resilin type (further discussed below), that displays both LCST and UCST and reversible gelling [151].

**Figure 10:**
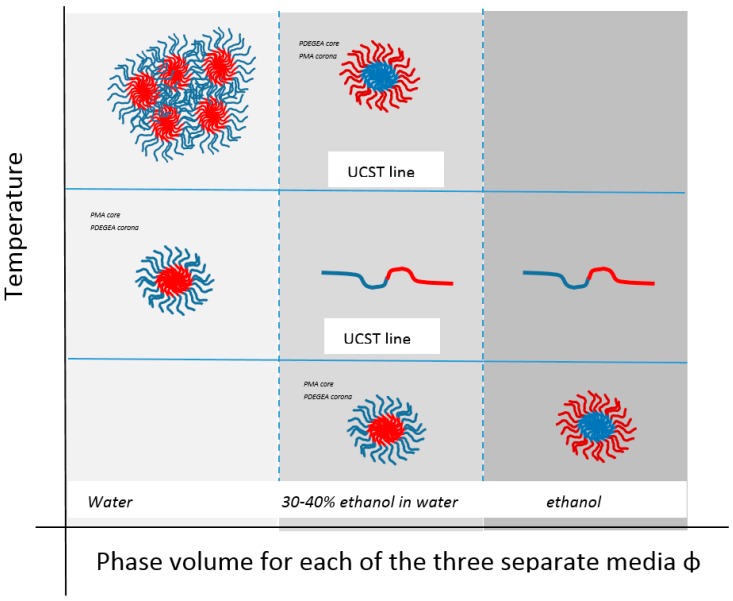
Schizophrenic (reverse) PDEGEA-PMA micelles in ethanolic solution. Adapted from [152].

Micellar systems can also be found that do this. For binary solvent micellar systems, Zhang discussed some block copolymers of 2-phenyloxazoline (PhOx, hydrophobic) and methyl (or ethyl) oxazoline (MeOx or EtOx hydrophilic) incorporated into the copolymer in gradient form [96]. One of these, PhOx_50_ EtOx_50_ in ethanol water 40:60 mix, produced a micellar system that is an example of a material exhibiting both UCST and LCST that was fully reversible. Fine-tuning of an aqueous alcohol binary solvation of poly(diethylene glycol ethyl ether acrylate)-poly(methyl acrylate) (PDEGEA-PMA) block polymers has been shown by Can to produce so-called schizophrenic systems that not only have both LCST and UCST behaviour but, in the process, also exhibit a micelle form reversal where the PMA component can be in either core or corona [152] (Figure 10). Zhang reports a similar but aqueous system [153], while Shih has produced hemocompatible micelles of poly(*N*-isopropylacrylamide)-*block*-poly(sulfobetaine methacrylate) (PNIPAAm-*b*-PSBMA) block copolymers using ATRP, such that they were non-fouling and anticoagulant between 4 and 40 °C and had potential in blood-contacting roles.

## 3. Hydrogels and Crosslinked Organogels Including Particulate Forms

Hydrogels are three-dimensional, elastic lattices of hydrophilic polymer, absorbing rather than dissolving or dispersing in water, the swelling capacity depending on the detail of the crosslinks and components. Many natural and synthetic polymers can be used to form hydrogels which by definition are aqueous [154]. They can be non-ionic, ionic or zwitterionic [155]. Some authors have included non-covalently stabilised semi-solid materials in this class and thus it might embrace gelatin, alginate, pectin and agarose if not a wider range of less elastic mucilages [156]. However, others would restrict the definition to covalently stabilised structures on the basis that otherwise the solvent-induced swelling properties that are usually associated with the immersion of hydrogels would ultimately lead to molecular dispersion via an irreversible gel to sol process. The limitation of this restriction is in the production of covalently bonded micro- to nanogels that do disperse (but not dissolve—see below); however, this review assumes hydrogels have permanent bonding. Advantages of a covalently stabilised structure in pharmaceutical formulation is in the protection of drug molecules, the imposition of both simple and complex release kinetics and the possibility of building phase volume change as a stimulus response, rather than gel to sol change as in dynamic bonding. Hydrogels, like their linear counterparts, comprise component monomers with a range of properties allowing tailoring, but in addition have multipurposed crosslinking agents so that the end product can have bespoke responsiveness in a robust system. One range of continuing innovative copolymeric hydrogels comprises a degree of hydrophobicity associated with the monomeric complement, for example using particular acrylics and also derivatives of amino acids such as valine [157], lipids such as cholesterol [158] and semi-synthetic polysaccharides such as chitosan adjuncts, starch [159] and modified cellulosics [160]. These can impose a volume-phase transition temperature (VPTT) on the hydrogel analogous to the critical solution phase temperature of a monomeric system.

**Figure 11:**
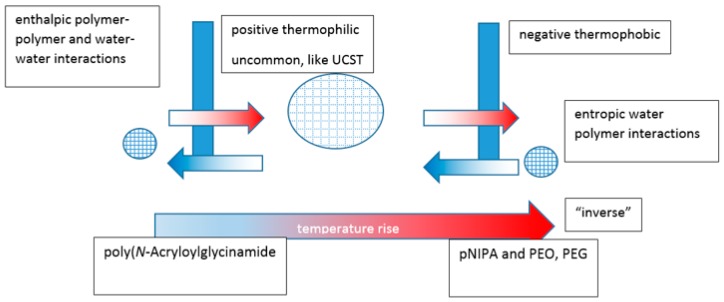
Swelling characteristics of hydrogels.

In a review by Mah, crosslinked hydrogels comprising monomers with UCST characteristics contract on cooling below the VPTT relating to the monomeric UCST [161]. They swell on heating above it and are known as positive thermophilic or positive temperature-sensitive hydrogels. Attempts have been made to model temperature-induced unconstrained and constrained swelling [162]. Negative thermophobic or negative temperature-sensitive hydrogels, like crosslinked PNIPAm, contract on heating above the VPTT relating to the LCST and swell below it. For hydrogels, contraction happens with the exclusion of water and the expansions require a source of water to occur. The volume changes are consistent with the globule to coil to globule theoretical continuum of the monomeric systems, with the analogous enthalpic and entropic driving forces (Figure 11). An aqueous hydrogel system rarely if ever demonstrates both types of VPTT although, as described above, there are examples of dual critical solution temperatures for linear and branched equivalent structures.

Transitions of this sort can be smooth and continuous or sharp. Much depends on transient bonds between polymer chains and polymer-solvent bonds, such as H-bonding, rather than the covalent bonds holding the hydrogel structure together [161]. Hydrogels can be categorised in various ways. They can be manufactured as block and graft types, each with a variety of cross-linker molecules. Graft copolymers perform most rapidly, particularly comb-like versions [163], and any one of the monomeric components can be thermoresponsive although thermophilic behaviour is quite difficult to engineer because of the effect of polymerisation on water solubility of the monomer.

Temperature-induced shrinking, as with covalently crosslinked PNIPAm hydrogels, is found for many materials with additions to the category including, for example, poly(*N*-acroyl-*N*-propyl piperazine) polymers [164]. Casolaro describes cobalt and ferrous magnetic nanoparticles incorporated into thermoresponsive vinyl hydrogels, based on the α-amino-acid residues phenylalanine and valine, to deliver doxorubicin into HeLa cells [165]. There are also other similar cases for crosslinked magnetic nanogels [166,167].

Hydrogels can be constructed of the one homo or copolymer network but it is also possible to build more than one network that coexist in the same volume (interpenetrating networks or IPNs). Alzari compared thermoresponsive terpolymers with interpenetrating networks, comprising *N*-isopropylacrylamide (NIPAm), hydroxyethyl methacrylate (HEMA), 2-acrylamido-2-methylpropane sulfonic acid (AMPSA) copolymerised with varying ratios of the latter two. The IPNS were synthesised by two methods, including frontal polymerisation. The thermoresponsive swelling was quite different for the two, with the high AMPSA concentration terpolymers swelling strongly above a UCST (which seems to be wrongly ascribed to thermophobic behaviour). Some IPNs were unresponsive irrespective of concentration, but others shrank presumably with LCST characteristics though only if the HEMA-AMPSA network was pre-synthesised and the PNIPAm polymerised in situ i.e., the reverse method did not perform this way [45]. A cyclically thermophobic (swells with cooling) semi-IPN of starch within a PNIPAm structure was reported by Dragan [168] as a system to release diclofenac sodium in temperature-dependent pulses that were additionally influenced by pH. It was postulated that these might be useful for pyrexic spikes because of the apposite temperature range of the system. The cyclodextrin (CD)-enhanced drug loading of ibuprofen and the 35 °C responsive zone was proposed as a tool for pulsed delivery, for a similar proposed purpose by Wang [169]. A superficially similar theme is to be found in work by Wei [170], who investigated injections of a CD polymer to provide sustained release in a rabbit arthritis model. This, however, is not a hydrogel but a micellar poly(ε-caprolactone)-poly(ethylene glycol)-poly(ε-caprolactone) copolymer (PCEC-β-CD), for which the CD was host to indometacin. A further difference between this and Wang’s work is that this is an essentially LCST system where the micelles gel as previously described when raised to body temperature and is thus not like the UCST ibuprofen hydrogel for which additional release might be triggered [169]. Wang also reported a positively responsive (thermophilic i.e., shrinking with cooling and thus relating to UCST) hydrogel synthesised by grafting poly(acrylic acid) (PAAc) to maleic anhydride—cyclodextrin (MAH-β-CD) and forming an IPN with polyacrylamide (PAAm). Schmidt [171] summarises the incorporation of CD into many structure types including hydrogels and micelles, whereby they can temper the shape and content as well as crystallinity of the polymer being synthesised by providing protective in roles as mediators in polymerisation or post-polymerisation. For hydrogels, CDs can be used as crosslinkers if the accepting group fits. In fact, fit is possible for multiguest accommodation. In the last decade, many papers have built on this CD crosslinking concept including an injectable, bioadhesive complex with gelatin and a pH and temperature-responsive type [172,173]. A superbly graphically illustrated review by Arunachaleam [174] considers cyclodextrin and polyrotaxane structures in responsive gels. Polyrotaxanes comprise one or more circular host molecules, such as a cyclodextrin, threaded onto a dumbbell-shaped linear backbone, so that the host molecule cannot escape. Complex construction is possible and the variety is huge. Some representatives of the group are thermoresponsive gels, such as a hydrogel formed from dibenzylammonium-terminated linear polycaprolactones crosslinked in this way with a tetravalent DB24C8 crown-bearing structure.

In general, some of the applications for such hydrogels can involve tissue interaction and the disadvantages of covalent systems are in the potency of any unbound multifunctional monomers in terms of biotoxicity and reactivity with the matrix content. Hennink has reviewed hydrogel synthesis methodology with particular reference to this aspect and also to implants of proteins which are themselves vulnerable if incorporated during covalent bond formation or before the unreacted monomer is removed. However, the subject, as was the case for micelles, is fast developing [7,175,176]. Where delivery or other incorporation of protein molecules is involved, protein to pore size ratio is often important. Either gradual hydrogel degradation can be or protein diffusion can be required, and these can be stimulus responsive including to temperature [177,178,179,180]. This implies that parts of the network might need to be labile so that local conditions can foster hydrolytic processes to proceed either chemically or by enzyme action to slowly release the protein payload and preferably to produce harmless excretable products.

Similar apolar (crosslinked organogel) systems can be formed. For example, some are mesoporous such as one described by Helgeson formed from thermogelling of nanoemulsions. The gelling here can be a reversible process or, using crosslinkable gelators, can be photo-polymerised and in some cases converted into a strong but soft material with permanent nanoporous characteristics [181]. A crosslinked valine-containing pH-sensitive hydrogel has been fabricated from an organogel intermediate [182]. Sagiri discusses bigels defined here as a high temperature emulsification of a gelatin hydrogel and a stearic acid-based organogel of sesame oil or soybean oil. The bigels are said to have aggregates whereas superficially similar emugels were true, finely dispersed droplets in the continuum. However, although these showed endothermic peaks at 44 and 85 °C, no claim was made for thermoresponsiveness [80]. A similar system has recently been described by Singh [183,184].

## 4. Proteins

Proteins are also thermosensitive and capable of sol-gel change although the mechanisms involved are less clear cut with folded active sites formed and combinations of covalent and non-covalent bonds. The distinction between polymer gels and particulate gels (e.g., metallic hydroxides) finds a resonance with protein gels that have occurred via aggregation [185,186,187]. The protein gelatin is exceptional in many ways, acting more like some polysaccharides (e.g., carrageenan, agar, agarose) reversibly melting rather than irreversibly unfolding and coagulating with increased temperature like myofibrillar and ovalbumins. The sol formation for gelatin is a denaturation process and the helical formation is part of its remarkable refolding. In general, gelation of proteins more often involves irreversible disulphide bond rupture. Gelatin is again unusual in that it has none, probably contributing to the reversibility of its structure compared with others in which the thermoresponsive utility is limited. Gelatin itself forms a physically entangled gel, but recently, Yue built on a wealth of work where gelatin has been methacrylated to give thermoresponsive gels with a wide range of mechanical properties and fabricated products [188]. Heating proteins more often results in a different behaviour, with molecular changes on several levels, affecting tertiary and secondary structure. Unfolding at the endothermic denaturation point is entropy driven and as remaining crystalline regions melt, a mobile amorphous state may then, unlike the gelatin case, foster interactions that then cause irreversible gelling at a transition possibly identical to an LCST [99]. This follows the formation of apolar aggregates which may themselves be disulphide-stabilised from free thiols or lysine amine groups. Artificial, biotechnically formed elastin-type polypeptide structures with the repeating unit Valine-Proline-Glycine-Xaa-Glycine, where Xaa represents a non-proline “guest” amino acid, mimic natural analogues throughout the biological world. These materials exhibit phase transition behaviours that are specific to structure but are triggered by stimuli, including temperature and pH, and thus they share the characteristics previously discussed. When triggered, for example by temperature, insolubility and aggregation occur that involve a helical interaction of molecules with hydrophobic bonding. This property can be conferred on a conjugate used, for example, in ligand sensing, movement, biochemical processes, drug delivery, etc. In terms of the latter, it can be honed to a temperature differential said to distinguish some tumour cells, thus allowing cytoplasmic retention of drugs conjugated to the peptide polymeric structure [189,190]. Similar properties can be found in silk-elastin-like proteins (SELPs) for which Xia has proposed production methodology [191]. Preliminary work with these materials produced materials where the aggregation was non-reversible. In recent work, Glassman used valine as the principal guest amino acid and arrested the spinodal separation process previously leading to the heat-induced coacervation characteristic of the class. The process can be controlled and produces reversibly thermoresponsive materials that can be toughened for use in biomedical settings among others [192,193].

## 5. Thermoresponsive Vitrimers and Composites

In contrast to most hydrogels, covalent thermal curing can be engineered, such as for conventional thermosetting polymers exemplified by polyurethane-forming components bearing functional groups. These have mechanical strengths that generally far outweigh those of even the toughest types of hydrogels [194]. However, they are also resistant to solvents so they represent the opposite end of a spectrum of polymer types that would find little application in diffusional drug delivery systems like implants and are themselves outside the scope of this review.

However, an intermediate type of material has been developed comparatively recently—vitrimers i.e., a polymer that has aspects of vitrification [195]. These have been described as mechanically strong but, when heated, they have gradually active associative exchange mechanisms [196] that occur over a wide quasi-glass transition temperature range, thus combining features of thermoplastic and thermosetting materials [197,198,199]. This means that they have the same characteristics and can be self-healing and also be reshaped, which has implications for sustainability and also for recycling [15,200,201]. Fortman describes poly(hydroxyurethanes) that can be activated by heat and stress and proposes their utility on the basis of low toxicity and catalyst-free reparability [202]. A medical example is a heat-activated aqueous solution that creates nanomolecular bridging of tissue via a gelling process similar to medical cyanoacrylates, but without the solidity or the sting. Popular press science articles refer to this being relevant to situations where suturing is impractical. Interestingly, an all-DNA vitrimer-like material has been described in which star-shaped sequences of designer single-stranded DNA bind via sticky ends to network upon cooling and can be used as encapsulation devices. These are more fragile than many synthetic vitrimer types, but still have the property of continual self-healing with parameters that can be tuned catalytically [203].

The remaining thermoresponsive polymer category in this review are composites with shape memory [204] imposed by forming above a transition temperature T_trans_ that is greater than the glass transition (Tg) or the melting temperatures (Tm). These elastic compounds are typically improved in terms of aggregation, strength and thermoresponsiveness by adding nanosized rods, films and fibres of metal oxides, graphine and other carbon materials, noble metals and natural polymers such as cellulose. Such materials are useful in many settings, as discussed by Pilate, including smart medical devices [205]. In many envisaged uses, external heating would be less convenient than internally generated heat using the fillers to convert other energy forms, such as light, electrical, magnetic, chemical or acoustic. Carbon black, for example, dispersed through the chosen polymer gels [206] converts electrical energy to heat and creates the shape change which is temporary provided the temperature does not exceed T_trans_ [205]. A synergistic activity between carbon black, carbon nanofibres and nickel strands, whereby electrical connectivity was enhanced, optimised the heat transfer that underlies the reversible contractile behaviour of proposed artificial muscles [207]. In a variant, where gold nanorods were incorporated into poly(sodium 4-styrenesulphonate), the temperature rise was induced by near-infrared light which can penetrate tissue and was able to trigger the release of doxorubicin conjugated to the gold while a gold nanocage-hyaluronic acid platform was similarly photothermally employed [208,209]. Wang has a somewhat similar system to deliver plasmid-derived genetic material to the cytosol from endosomes via gold nanorods exposed to photothermal excitation at 808 nm [210].

## 6. Concluding Remarks

Thermoresponsive materials have been the subject of many published studies and have been reviewed regularly, reflecting the explosion in interesting and complex molecular structures such as the synthetic methodology, specifically Reversible Addition Fragmentation chain Transfer (RAFT) and Atomic Transfer Radical Polymerisation (ATRP). The focus in this review has been the viscosity changes as these polymeric molecules respond to temperature change, with the accent on upper and lower critical solution temperatures (UCST and LCST). Where possible, these have been applied to biomedical utility, especially drug delivery, medical sensing and the combination of the two for smart systems wherein sensing and actuation are combined. Lessons can be learned from the use of such materials in other fields where they exhibit UCST, LCST and related behaviour, using low molecular weight gelators, single polymeric molecules and their aggregates. There is much published on the LCST behaviour and its possible exploitation for medicine. Unusually, we have explored UCST materials more fully because the area has been sparse and yet there may be a rich seam to be mined in organogels and self-assembled hydrophobic materials such as micelles.

Covalently stabilised gels give further opportunity for actuation both mechanically and by combination with other inputs (pH etc.) and outputs (thermochromic, conductive etc.) and, given that acceptable pharmacologically active molecules are scarce, the use of novel developments in thermoresponsive materials is poised to enable targeting and activation in fields such as cancer, diabetes and immune-provoked inflammatory disease. The dream of a continuously responding dose form for mutational change is a distant but achievable goal.

There are, however, concerns about safety mostly in the areas of toxic leaching of monomers, failure to degrade, inflammatory tissue response and infection. The concepts of precision of dose rate, reproducibility of short and long timescales and dose dumping are also important in this regard, especially for the delivery of potent materials. In this review, we have also briefly addressed the porous behaviour of permanent gels including the relevance of pore size to solute ratio, enzyme vulnerability design and tissue stresses on block implants. We have also contextualised the design and use of particulates and of self-healing, shape-adjusting materials that have dynamic covalent bonding intermediates between the permanence of covalent bonding and the transience of self-assembly.
